# Classification of colon cancer patients into consensus molecular subtypes using support vector machines

**DOI:** 10.55730/1300-0152.2674

**Published:** 2023-12-15

**Authors:** Necla KOÇHAN, Barış Emre DAYANÇ

**Affiliations:** 1 Department of Mathematics, İzmir University of Economics, İzmir, Turkiye; 2 İzmir Biomedicine and Genome Center, İzmir, Turkiye; 3 Basic Medical Sciences, Faculty of Medicine, İzmir University of Economics, İzmir, Turkiye

**Keywords:** RNA-seq, colon cancer, classification, support vector machines

## Abstract

**Background/aim:**

The molecular heterogeneity of colon cancer has made classification of tumors a requirement for effective treatment. One of the approaches for molecular subtyping of colon cancer patients is the consensus molecular subtypes (CMS), developed by the Colorectal Cancer Subtyping Consortium. CMS-specific RNA-Seq-dependent classification approaches are recent, with relatively low sensitivity and specificity. In this study, we aimed to classify patients into CMS groups using their RNA-seq profiles.

**Materials and methods:**

We first identified subtype-specific and survival-associated genes using the Fuzzy C-Means algorithm and log-rank test. We then classified patients using support vector machines with backward elimination methodology.

**Results:**

We optimized RNA-seq-based classification using 25 genes with a minimum classification error rate. In this study, we reported the classification performance using precision, sensitivity, specificity, false discovery rate, and balanced accuracy metrics.

**Conclusion:**

We present a gene list for colon cancer classification with minimum classification error rates and observed the lowest sensitivity but the highest specificity with CMS3-associated genes, which significantly differed due to the low number of patients in the clinic for this group.

## 1. Introduction

Colon cancer is one of the most common cancer types worldwide and the second and third leading cause of cancer deaths for men and women, respectively. Approximately 8% of cancer-related deaths in the world are associated with colon cancer (Schweiger et al., 2013). The molecular heterogeneity and complexity of this type of cancer make the prediction of the disease and potential treatments more difficult. To better characterize and resolve heterogeneity, researchers have focused on the subtyping of colon tumors. The Colorectal Cancer Subtyping Consortium (CRCSC) published a study in which CRC patients were stratified into 4 distinct Consensus Molecular Subtypes (CMS) and 1 unknown group in which patients had no CMS information (no label) (Guinney et al., 2015).

For CMS subtyping, CRCSC developed the CMSclassifier algorithm, which uses hundreds of genes from all available genome data (Buechler et al., 2020). Subsequently, an R package called **CMScaller** was developed and published by Eide et al. (2017) that uses more than 500 genes from the genome data. However, before Buechler et al. published RNA-Seq and microarray data-based CMS subtyping (ColoType) with 40 genes (Buechler et al., 2020), there had been no specific RNA-Seq-based CMS approach. The rationale for this study is that microarray and RNA sequencing technologies are inherently different, and both technologies have some shortcomings—as summarized in Eilertsen et al.’s study (2020)—such as inherent technical biases observed with microarrays related to cross-hybridization and limited dynamic range of expression (Wang et al., 2009). These shortcomings impact subtype distributions according to clinically relevant classification frameworks such as CMS. As long as the systematic biases are addressed (representation of short genes and genes with low expression levels), RNA-Seq is a reliable and preferred method for transcriptomic subtyping of colon cancer by whole transcriptome profiling (Wang et al., 2009).

Studies show that cancer classification based on gene expression data has become an important part of modern medicine. Therefore, in this study, we applied support vector machines (SVMs) to classify CRC patients with CMS status based on their gene expression levels. We mainly focused on RNA-Seq data that includes CMS information for each patient.

## 2. Materials and methods

### 2.1. Gene expression data and survival data

RNA-Seq data for the CRC patients were obtained from the TCGA database using the **TCGAbiolinks** package (Colaprico et al., 2016). We considered patients with primary tumor (PT) and solid tissue normal (STN). Among these patients, we selected those who were diagnosed with primary adenocarcinoma but who had not received therapy. We used disease-specific survival (DSS) data for the survival analysis; the survival data of the TCGA COAD samples was obtained from Liu et al. (2018). To identify the molecular subtype-specific prognostic genes in colon cancer, we downloaded and used the subtype information of TCGA COAD patients from synapse.org. After collecting the required information, we were left with 29 patients in CMS1, 82 in CMS2, 27 in CMS3, and 58 in CMS4 (Table 1). We filtered the genes with very low or no expression using fragments per kilobase of transcript per million (FPKM) values. We filtered the genes with FPKM values below 0.5 in both PT and STN to avoid the systematic bias of RNA-Seq data on genes with low expression. After this filtering, 14,334 genes remained for further analysis. Following the filtering process, we used log-CPM(x+1) to normalize the raw counts to overcome any variations that might arise from experimental differences (Robinson and Oshlack, 2010; Jun et al., 2012; Ritchie et al., 2015).

### 2.2. Identification of subtype-specific prognostic genes for colon cancer using FCM

**Figure 1:**
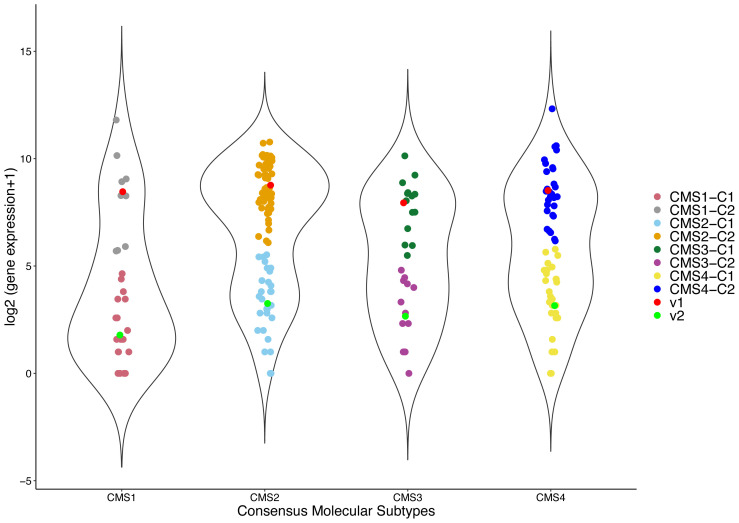
FCM clustering. Stratification using FCM, where C1 and C2 are cluster centers for cluster I and II, respectively; points in the same cluster are similar, and points that overlap are assigned to one of the clusters with respect to the maximum membership degrees.

To identify subtype-specific prognostic genes, analyses were performed separately for each CRC molecular subtype. The FCM clustering algorithm was then applied to stratify patients into 2 clusters (groups) with membership degrees for each patient and cluster centers. The algorithm assigned each patient to one of the clusters with the maximum membership degree, which displays the degree of belonging to the corresponding cluster. A representative FCM clustering of the FOXJ1 gene for each subtype was depicted as a violin plot, as shown in Figure 1.

The genes that could significantly be differentiated between the survivals of these 2 groups were chosen with a cutoff p-value of 0.01 using log2 expression values. By applying an FCM-based approach, we obtained 86 genes for CMS1, 148 genes for CMS2, 8 genes for CMS3, and 53 genes for CMS4, all statistically significant (p < 0.01). After reducing the number of genes, we performed univariate Cox regression to obtain the most informative genes for further analysis. As a result of univariate Cox regression, we reduced the numbers to 6, 48, 2, and 25 for CMS1, CMS2, CMS3, and CMS4, respectively. It should be noted here that since we could not find any significant genes for CMS3 with a maximum p-value of 0.01, we considered the p-value cut-off to be 0.05 for the CMS3 group to have at least 1 gene for each molecular subtype.

### 2.3. Gene selection

With the advent of next-generation sequencing, it is possible to detect tens of thousands of genes simultaneously, providing deep insight into cancer classification problems. The major challenge in classifying gene expression data is to extract disease/cancer-related information from a large amount of redundant information and noise. Therefore, obtaining significant information is a key step in classifying gene expression data.

**Figure 2:**
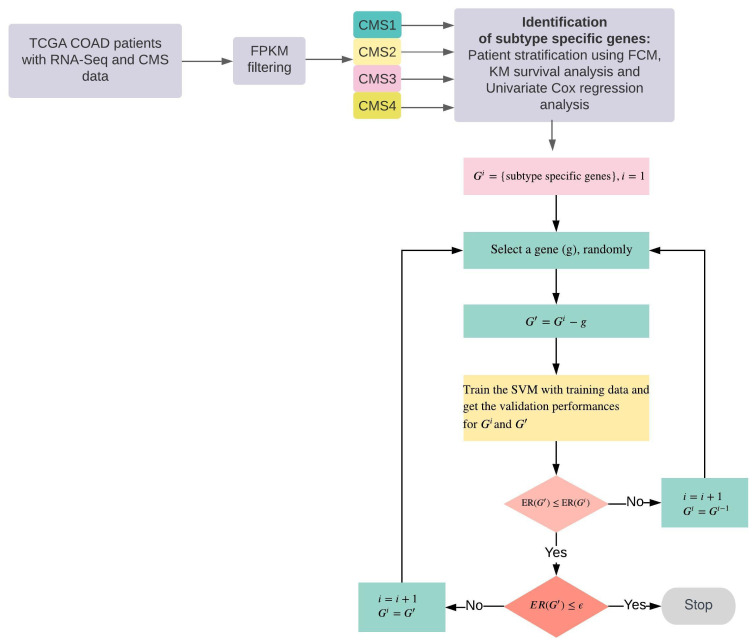
Flowchart of the study’s method. Identification of prognostic genes and backward elimination algorithm for gene selection to classify CRC patients. ER: error rate; G: the gene set.

Rather than starting with more than 20,000 genes and applying any feature selection methods, we began with the molecular subtype-specific prognostic genes identified in the previous section. These genes play a crucial role in colorectal cancer subtype classification. We searched for the gene list using a backward elimination method (Figure 2).

### 2.4. SVM classification

SVMs are kernel-based machine-learning algorithms developed by Vapnik (2000). They have been applied to numerous areas, such as pattern recognition, medicine, bioinformatics, biological studies, and other sciences.

An SVM finds an ideal decision boundary called an ideal separation hyperplane to separate classes. The ideal decision boundary or hyperplane is determined according to the maximum margin principle. The algorithm locates the ideal hyperplane that maximizes the distance between classes. The vectors that define these hyperplanes are called support vectors.

If the classes are linearly separable, the SVM performs efficiently and splits the classes without an overlap. However, a perfect separation may not be observed in many real-life data sets. In that case, the SVM searches for the hyperplane, which minimizes the classification error rate and maximizes the margin (Bishop, 2006). If the data is linearly nonseparable, the SVM uses kernel functions (i.e. linear, nonlinear sigmoid functions) and radial basis kernels to convert nonseparable data into a linearly separable data form.

Cancer classification based on gene expression data has become an important part of modern medicine, providing an objective and accurate diagnosis of different types of cancers/diseases. A number of machine learning approaches, e.g., SVMs, random forest, and k-nearest neighbor, have been applied to gene expression data classification. However, these approaches pose challenges since patient tumors are not classified through gene expression but via pathological information in the clinical setting. This shows that there is a gap in the literature in terms of cancer classification at the gene expression level. Colon cancer is a type of cancer that requires further investigation. Therefore, in this study, we applied the SVM algorithm to the classification of colorectal cancer patients, as it is one of the most powerful supervised learning algorithms. The SVM is performed using the “**e1071**” package in R with a radial basis kernel and 10-fold cross-validation to optimize the model parameter.

### 2.5. Performance evaluation metrics

The class-specific performances were calculated according to the precision, sensitivity, specificity, false discovery rate (FDR), and balanced accuracy, defined as follows:


$$\begin{array}{l} \text{Precision}=\frac{\text{TP}}{\text{TP}+\text{FP}}\\ \text{Sensitivity}=\frac{\text{TP}}{\text{TP}+\text{FN}}\\ \text{Specificity}=\frac{\text{TP}}{\text{TP}+\text{FN}}\\ \text{FDR}=\frac{\text{FP}}{\text{FP}+\text{TP}}\\ \text{Balanced accuracy}=\frac{\text{sensitivity}+\text{specificity}}{2}, \end{array}$$

where TP represents true positives, FP represents false positives, TN is true negatives, and FN is false negatives. Overall performance is measured by the classification error rate (CER), shown below:


$$\text{CER}=\frac{\#\;\text{of misclassfied patients}}{\#\;\text{of patients in the test set}}$$

### 2.6. Statistical analysis

Statistical analyses were performed using R language (v.4.0.2). Kaplan–Meier and log-rank tests were performed to assess survival differences between clusters and risk groups. Univariate Cox regression analysis was performed using “survival” and “survminer” packages in R; p-values below 0.01 were considered statistically significant for all comparisons except for CMS3 subtype-specific prognostic genes, as previously described.

## 3. Results

We considered the discovery set used in identifying the subtype-specific prognostic genes as the training set and the test set as the TCGA COAD data not included in the training set. The CMS clinical information of all patients (training and test sets) was downloaded from synapse.org. The training set (66% of the dataset) was used to train the SVM classification model, and the test set (34% of the dataset) was used to measure the CER.

**Figure 3:**
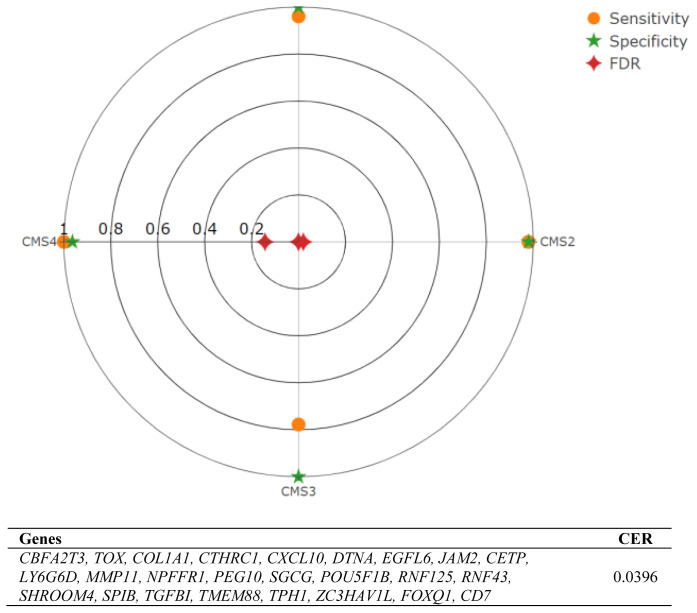
Performance analysis using 25 genes. Classification performances of each CMS in terms of sensitivity, specificity, and false discovery rate. Overall performance is given in terms of classification error rate, which is the ratio of misclassified patients over all patients in the test set.

The results show that when we used 25 genes, we reached the minimum CER, which is 0.0396 (Figure 3). Moreover, the subtype-specific statistics are given in terms of precision, specificity, sensitivity, FDR, and balanced accuracy (Table 2). We observed that CMS3 has the smallest sensitivity but the highest specificity, and the other subtypes (CMS1, CMS2, and CMS4) not only have high sensitivity but also high specificity for the 25-gene list.

## 4. Discussion

In this study, we discovered 2 gene lists for colon cancer classification with minimum CERs. The SVM is a kernel-based algorithm and one of the most widely applied classification algorithms in bioinformatics due to its high accuracy (Zhi et al., 2018). This is the first study to classify TCGA COAD patients using a new pipeline that involves identifying survival-associated genes and applying SVMs with backward elimination. Utilizing this novel method, we aimed to improve classification accuracy and identify potential prognostic biomarkers for colon cancer.

Molecular mechanisms have become increasingly important in the development of CRC. By combining molecular mechanisms with machine learning, we can deepen our understanding of what causes CRC and potentially find new treatment methods. Zhou et al. (2022) discovered prognostic markers for CRC by constructing molecular subtypes. The authors used different clustering methodologies to find markers that predict survival. Our approach differs from theirs because we used consensus subtypes and identified subtype-specific markers that predict survival. More precisely, we applied FCM clustering to identify 2 distinct groups with significantly differing survival characteristics.

CMSs of colorectal cancer patients are determined by molecular tumor pathologic information. Although patient treatment modalities for colon cancer today are prescribed by tumor staging, very few tools have been used to guide clinical decisions until now (Ågesen et al., 2012; Sveen et al., 2012; Shinto et al., 2020) Buechler et al. (2020) developed the 40-gene ColoType risk score model for CRC patient classification with an 88% performance in TCGA-COAD RNA-Seq data. To compare our results with that study, we used the 40 genes reported in their study in our training and test sets using the SVM classification algorithm. The error rate was measured as 0.13 when the 40 genes were used with our test set.

It is important to note that this study is specific to CRC RNA-Seq data with additional CMS information—that is, patients without CMS information were excluded. Thus, our approach is limited only to publicly available TCGA COAD data. We tested a single cohort; therefore, other cohorts with CMS information could be further investigated.

In order to identify CMS-specific genes, we considered patients who had primary adenocarcinoma and had received no prior treatment. We selected patients with DSS clinical information presented in Liu et al.’s study (2018) and whose DSS was more specific than “overall survival” (OS). Due to the low number of CMS3 patients in the TCGA COAD study and as our patient selection criteria further limited the number of patients in each molecular subtype, the patient number in the CMS3 group was relatively low (36). The reliability of this study could be further improved by using and combining more RNA-Seq gene expression data and patients’ CMS subtype information.

## 5. Conclusion

We identified a gene list for precise colon cancer classification, minimizing classification errors. Our results show the lowest sensitivity but the highest specificity when using CMS3-associated genes. This discovery is significant due to the limited number of patients in this clinical subgroup.

## Figures and Tables

**Table 1:** Data description. Number of patients used for the training dataset and test dataset.

	CMS1	CMS2	CMS3	CMS4	Total
Training set	28	82	27	58	195
Test set	125	49	9	18	101
Total	53	131	36	76	296

**Table 2:** Classification performance. 25 genes were used, leading to a minimal classification error rate.

	Precision	Sensitivity	Specificity	FDR	Balanced accuracy
CMS1	1.0000	0.9600	1.0000	0.0000	0.9800
CMS2	0.9796	0.9796	0.9800	0.0204	0.9798
CMS3	1.0000	0.7778	1.0000	0.0000	0.8889
CMS4	0.8571	1.0000	0.9634	0.1429	0.9817
